# Immunosuppressive therapy with rituximab in common variable immunodeficiency

**DOI:** 10.1186/s12948-019-0113-3

**Published:** 2019-05-06

**Authors:** Antonio Pecoraro, Ludovica Crescenzi, Maria Rosaria Galdiero, Giancarlo Marone, Felice Rivellese, Francesca Wanda Rossi, Amato de Paulis, Arturo Genovese, Giuseppe Spadaro

**Affiliations:** 1 Department of Translational Medical Sciences and Center for Basic and Clinical Immunology Research (CISI), World Allergy Organization (WAO) Center of Excellence, University of Naples Federico II, Via S. Pansini 5, 80131 Naples, Italy; 2 Department of Public Health, University of Naples Federico II, Naples, Italy; 3 Monaldi Hospital Pharmacy, Naples, Italy; 4 Centre for Experimental Medicine and Rheumatology, William Harvey Research Institute, Barts and The London School of Medicine and Dentistry, Queen Mary University of London, London, UK

**Keywords:** Common variable immunodeficiency, Antibody deficiency, Autoimmune cytopenias, Granulomatous lymphocytic interstitial lung disease, Rituximab, Anti-CD20

## Abstract

Common variable immunodeficiency (CVID) is the most frequent symptomatic primary antibody deficiency in adulthood and is characterized by the marked reduction of IgG and IgA serum levels. Thanks to the successful use of polyvalent immunoglobulin replacement therapy to treat and prevent recurrent infections, non-infectious complications, including autoimmunity, polyclonal lymphoproliferation and malignancies, have progressively become the major cause of morbidity and mortality in CVID patients. The management of these complications is particularly challenging, often requiring multiple lines of immunosuppressive treatments. Over the last 5–10 years, the anti-CD20 monoclonal antibody (i.e., rituximab) has been increasingly used for the treatment of both autoimmune and non-malignant lymphoproliferative manifestations associated with CVID. This review illustrates the evidence on the use of rituximab in CVID. For this purpose, first we discuss the mechanisms proposed for the rituximab mediated B-cell depletion; then, we analyze the literature data regarding the CVID-related complications for which rituximab has been used, focusing on autoimmune cytopenias, granulomatous lymphocytic interstitial lung disease (GLILD) and non-malignant lymphoproliferative syndromes. The cumulative data suggest that in the vast majority of the studies, rituximab has proven to be an effective and relatively safe therapeutic option. However, there are currently no data on the long-term efficacy and side effects of rituximab and other second-line therapeutic options. Further randomized controlled trials are needed to optimize the management strategies of non-infectious complications of CVID.

## Introduction

Rituximab (RTX) is a monoclonal antibody (IgG1k) that specifically targets the transmembrane protein CD20 expressed on pre-B and mature B lymphocytes [1–3]. Binding of RTX to its receptor results in significant depletion of B-cells in lymphoid tissues and peripheral blood by different mechanisms, including apoptosis, complement-dependent cytotoxicity, and antibody-dependent cytotoxicity [4–6]. Since hematopoietic stem cells do not express CD20, one course of treatment with rituximab is followed by B-cell repopulation of the peripheral blood starting usually within 6 to 9 months [7]. However, a subset of RTX-treated subjects develop prolonged B-cell deficiency and severe hypogammaglobulinemia requiring long-life immunoglobulin replacement [8–10].

RTX was first approved by the FDA in 1997 and by the EMA in 1998 for the treatment of relapsed or refractory, CD20-positive, B-cell, low-grade or follicular non-Hodgkin’s lymphoma [11]. In the following two decades, the use of RTX has progressively expanded to include, also with off-label indications, an increasing number of autoimmune diseases [12–15] (i.e., rheumatoid arthritis, anti-neutrophil cytoplasmic-associated vasculitis, systemic sclerosis, immune thrombocytopenia, etc.) and the EBV-related lymphoproliferative syndromes associated with bone marrow transplantation [16, 17].

Several studies reported the successful use of rituximab for the treatment of autoimmune and lymphoproliferative manifestations associated with primary immunodeficiencies, and in particular with common variable immunodeficiency (CVID) [18–20]. CVID is the most frequent severe antibody deficiency in adulthood and is characterized by the reduction of serum immunoglobulin levels (namely IgG and IgA) and the impairment of antibody production in response to pathogens and vaccines [21, 22]. This may be due either to an intrinsic defect of B-cell development or to a disrupted cross-talk between B and T cells [23, 24]. Beyond the impairment of B-cell functions, a number of other immune alterations have been described in CVID patients. Together, these contribute to the establishment of a complex immune dysregulation including naive T or NK deficiency, expansion of specific B-cell-subpopulations (i.e., CD21low and transitional B-cells), monocyte/macrophage activation, Th1 imbalance of T-helper follicular cells (TFH) associated with a IFN-γ driven inflammation and neutrophil-mediated T-cell suppression [25–35]. According to the heterogeneity of the immunological alterations, CVID patients presents a wide spectrum of clinical manifestations including infections, inflammatory and autoimmune diseases, and malignancies (cancer and lymphoma) [36–41]. The heterogeneity of the clinical picture makes CVID diagnosis challenging, thus contributing to the establishment of a significant diagnostic delay that affects both the long-term outcome and the quality of life of CVID patients [42–44]. Therefore, different population-based screening approaches have been recently introduced in the clinical practice to shorten diagnostic delay and improve long-term outcome [45, 46]. The mainstay of treatment of CVID is immunoglobulin replacement therapy (IgRT). Although immunoglobulin therapy is also used at higher dosage in a wide range of autoimmune and inflammatory conditions for its immunomodulatory effects [47], the main indication of this treatment is the lifelong replacement therapy of antibody deficiency to prevent and treat recurrent infections [48]. New immunoglobulin purification and stabilization methods have been developed thus allowing the administration of higher volumes and higher concentrations of immunoglobulins via both the intravenous and the subcutaneous route [49–55].

Thanks to the successful use of IgRT to treat bacterial infections, autoimmune diseases and malignancies have progressively become the major cause of morbidity and mortality in CVID patients [56–58]. CVID-related autoimmune and lymphoproliferative complications are poorly understood from the pathogenetic point of view and therefore difficult to manage [59]. Interestingly, in a large USA cohort the risk of death was 11 times higher for CVID patients with one or more non-infectious complication than for subjects who had infections only [56]. As consequence, a number of immunosuppressive protocols, which had been traditionally considered contraindicated in immunodeficiency patients over the last decades, have been increasingly used to treat autoimmune diseases and non-malignant lymphoproliferative manifestations associated with CVID [60, 61].

With the increasing evidence of effectiveness in several autoimmune diseases, also RTX has been progressively included in the therapeutic strategies for the non-infectious complications of CVID [19, 62–64]. Although the use of a B-cell depleting monoclonal antibody in a humoral immune deficiency may appear paradoxical, the heterogeneous pathogenesis of CVID, characterized by a complex immune dysregulation beyond the simple impairment of antibody production, could explain the potential efficacy of rituximab in CVID. Although no large randomized controlled trials have been conducted, several case reports, small series and cohort studies investigated the outcomes of RTX in CVID in last 15 years.

This review summarizes the evidence available on the use of rituximab in CVID. For this purpose, first we will discuss the mechanisms of action of RTX, focusing on the effects on the various immune cells; then, we will analyze the data available for the CVID-related complications for whom RTX has been used, namely autoimmune cytopenias, granulomatous lymphocytic interstitial lung disease (GLILD) and non-malignant lymphoproliferation.

### Mechanisms of action of rituximab

**Fig. 1:**
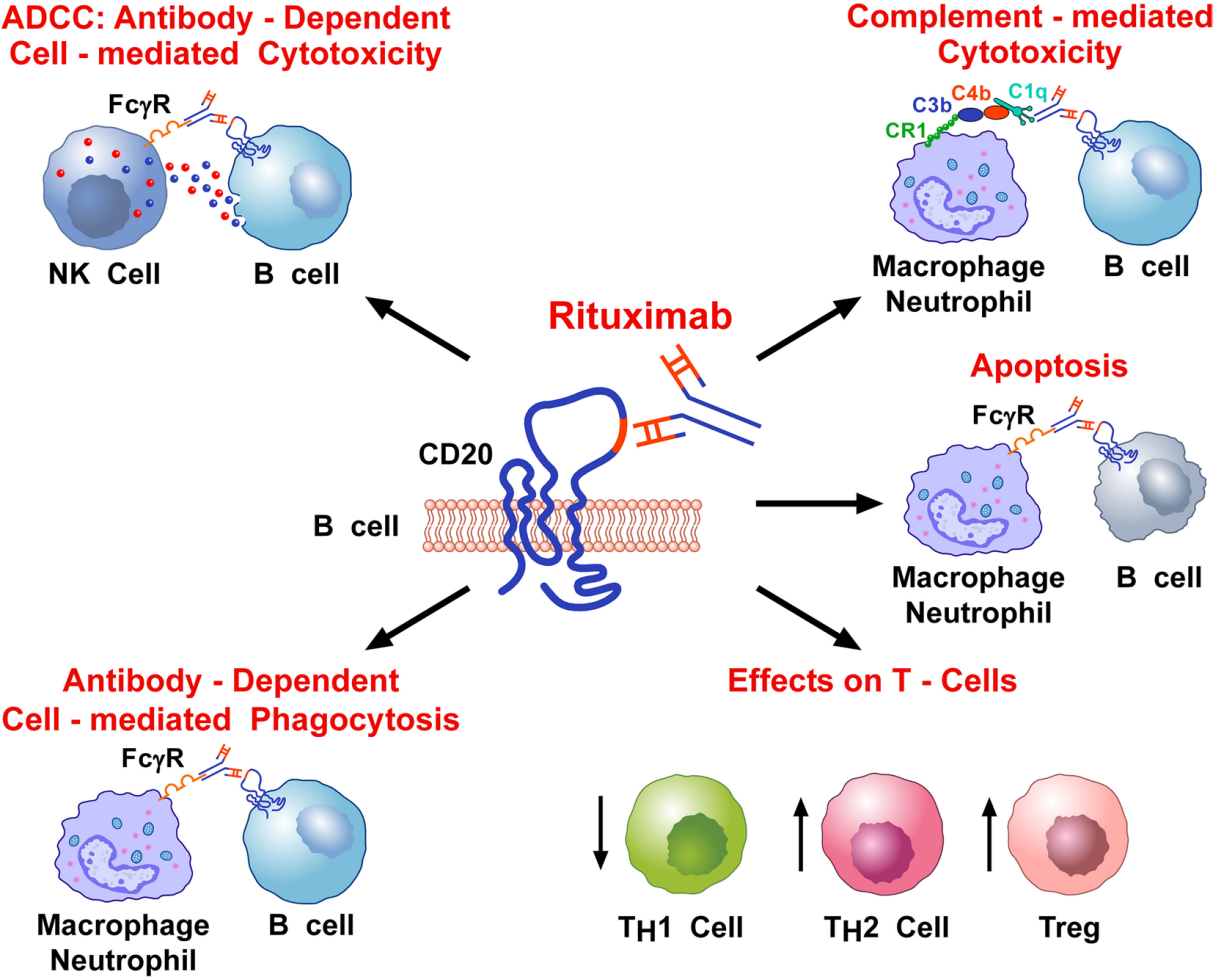
Schematic representation of the multiple mechanisms that have been proposed to explain the immunosuppressive effects of RTX. RTX is a chimeric IgG_1_ monoclonal antibody (mAb) (the Fc domain is humanized, whereas the Fab domain is murine) that targets the CD20 protein on human B-cells. RTX depletes B-cells primarily through an antibody-dependent cell-mediated cytotoxicity (ADCC) and complement-mediated cytotoxicity (CMC). ADCC occurs through the engagement of Fcγ receptor on NK cells by the Fc domain of RTX. Activated NK cells release perforin and granzymes that cause B-cell lysis. CMC occurs through the activation of the complement cascade by the interaction of RTX and CD20, the formation of anaphylatoxins (i.e., C5a, C3a) and the membrane attack complex. The Fcγ receptor is also expressed by human macrophages and neutrophils. Therefore, RTX can also cause antibody-dependent phagocytosis of B-cells by macrophages and neutrophils. Moreover, RTX can cause the apoptosis of B cells through the induction of both CD20 cross-linking on surface membrane and MAP kinase activation. Indeed, apoptotic B-cells are cleared by phagocytes, such as macrophages and neutrophils, through Fcγ receptor’s activation. Finally, there is some evidence that RTX can decrease Th1 cells and can increase Th2 and Treg cells

Multiple mechanisms have been proposed to explain the efficacy of RTX in autoimmune diseases and non-malignant lymphoproliferation. These effector mechanisms may act individually or collectively and are only in part ascribable to B-cell depletion. RTX depletes B cells primarily through three different mechanisms: antibody-dependent cell-mediated cytotoxicity (ADCC), complement-mediated cytotoxicity (CMC) and induction of apoptosis (Fig. 1) [65]. Among these, ADCC and CMC seem to be the most accountable for the B-cell depleting effect of RTX in vivo [66].

RTX is an IgG1 monoclonal antibody, which can activate the complement cascade, leading to 3 remarkable effects: (a) the generation of anaphylotoxins (e.g. C5a, C3a) that amplify the recruitment of effector cells; (b) the formation of a membrane attack complex (MAC) that results in the direct cell lysis; and (c) the liberation of cell cleavage fragments that act as opsonins [67, 68]. However, the differential depleting-effect of RTX to B-cells may depend on individual factors, as the expression level of the complement regulatory proteins CD46, CD55 and CD59 [69].

The relevance of ADCC for the in vivo response to RTX has been confirmed by the genetic analysis of Fc receptor polymorphisms in populations of treated patients, where FcγRIIIA-158 polymorphisms were shown to increase RTX-induced ADCC and improve clinical outcome [70]. This finding supports the hypothesis that the ADCC is the most important effect exerted by RTX [71, 72]. Consistent with this evidence, RTX was demonstrated to cause polarization of CD20, intercellular adhesion molecule 1 (ICAM-1), myosin, and the microtubule organizing center (MTOC), thus inducing the preferential cell-killing by effector NK cells [73]. However, several other immune cells, including polymorphonuclear cells and macrophages, express activating Fcγ receptors. Similar to ADCC, opsonized cells activate FcγRs on the phagocyte surface, resulting in an antibody-dependent cell-mediated phagocytosis (ADCP). Also, phagocytes may play an important role in the clearance of apoptotic B cells after RTX treatment [74].

B-cell depletion in peripheral blood after RTX treatment is higher than 99% [75]. However, this could not reflect the depletive effect in lymphoid tissues, where there can be considerable variations between individuals. A study evaluated the splenic immune responses in patients with immune thrombocytopenia (ITP) to address the immunomodulatory effects of RTX. The authors found marked follicular atrophy and a significant reduction in the number of splenic B lymphocytes in ITP patients treated with RTX. On the contrary, the number of plasma cells was increased in splenic samples from ITP patients who did not improve after RTX treatment [76]. The latter findings support the concept that the long-lived plasma cells, which are not affected by RTX, can sustain pathogenic antibody production for a variable time after RTX, thus being responsible for the poor or delayed clinical response. Of note, in the same individual the depletion’s grade tends to be similar in different tissues, suggesting that the extent of B-cell depletion achieved may be influenced by individual factors [77]. Among these, the antigen modulation, which refers to the CD20 endocytosis after binding the antibody, may play an important role, through the reduction of the recruitment of Fcγ receptors on effector immune cells [78].

Another cause of incomplete B-cell depletion is the induction of anti-RTX antibody response, which may influence drug action and clearance rates. This phenomenon has been described in patients with autoimmune diseases who received a RTX-retreatment. However, most large studies found no association between the presence of anti-RTX antibodies and clinical outcome [79, 80].

Even though the major part of RTX-immunomodulatory effects are accountable to B-cell depletion, evidence has proved that RTX also may target both helper and cytotoxic T-cells. The effect of RTX on T-cell compartment mainly consists in both the increase of T regulatory cells and the normalization of Th1/Th2 ratio, which is frequently increased in some autoimmune disorders as ITP. In a study in ITP patients, the major T-cell abnormalities observed were the increase of Th1/Th2 ratio, the over-expression of Fas ligand on Th1 and Th2 cells, the over-expression of Bcl-2 mRNA and the expansion of oligoclonal T cells in ITP patients compared to controls [14]. These abnormalities were reverted in RTX-responders after 3 and 6 months of treatment, whereas they remained unchanged in non-responders. Consistently with this findings, a significant increase in the Th1/Treg ratio was detected in splenic samples from ITP patients who failed RTX therapy compared with controls and to ITP patients not treated with RTX [76]. From a theoretical point of view, these results support the use of RTX in autoimmune and lymphoproliferative complications of CVID. Indeed, various studies have recently demonstrated a skewed memory CD4+ T-cell differentiation toward a CXCR3+CCR6− TH1 phenotype both in blood and lymph nodes in patients with CVID and immune dysregulation [28, 29].

### Autoimmune cytopenias

Cytopenias, including immune thrombocytopenia (ITP), autoimmune hemolytic anemia (AIHA) and autoimmune neutropenia (AN), are the most common hematological autoimmune disorders in CVID [81]. Autoimmune cytopenias (AC) may be the first evidence of immune dysregulation, preceding the occurrence of recurrent infections and even of hypogammaglobulinemia [82]. In the last report from the European CVID Registry by the European Society for Immunodeficiencies (ESID), AC were found to be correlated with enteropathy, granulomatous disease, splenomegaly and splenectomy, low IgA level, and later age of onset of CVID, while had been already associated with the reduction of IgG class-switched B-cells (CD27+IgM−IgD−) in peripheral blood [83, 84]. Although the pathogenesis of AC in CVID is not completely understood, various mechanisms have been proposed: the reduction of T regulatory (Treg) cells, whose relevance is supported by the finding of high rates of both granulomatous disease and autoimmunity in the subset of CVID patients with very low Treg cells [85, 86], and the persistent antigenic exposure to microbial antigens, which could trigger autoimmunity by molecular mimicry [87]. Recently, Romberg et al. found that patients with CVID and AC displayed irregularly shaped hyperplastic germinal centers with an increase in numbers of circulating TFH cells correlating with decreased regulatory T-cell frequencies and function [88].

ITP is the most common AC in CVID with a 7% to 14%, prevalence while the proportion of CVID patients with AIHA and AN is 4–7% and 1%, respectively [56]. ITP is also the most challenging AC to manage, often requiring immunosuppressive combination protocols and multiple treatments [89].

The standard treatment of CVID-associated AC consists in the use of glucocorticoids up to at 1 mg/kg prednisone-equivalent, associated, if a rapid response is required, to high-dose intravenous immunoglobulin (IVIG) (1–2 g/kg in 5 days). Rho (D) immunoglobulin is an option for non-splenectomized Rh-positive subjects who cannot receive glucocorticoid treatment [90]. Although the initial response rates are around the 85%, relapses are very frequent, thus requiring prolonged use of high-dose glucocorticoids [91–93]. Over the past decades, splenectomy has been the traditional second line approach in non-responders or relapsing patients and, behalf the introduction of RTX, up to the 50% of CVID-ITP patients required splenectomy as second-line treatment [19]. The largest multicenter retrospective study on splenectomy was carried out by the ESID in 2013 and reported the outcome of splenectomy in 45 CVID patients over a 4 decade period [94]. Splenectomy proved to be an effective long-term treatment in 75% of CVID patients with autoimmune cytopenia not worsening mortality.

RTX was first used for CVID-related AC, in 2004 [91]. After this report, various cases have described the outcomes of RTX in AC, reporting high response rates (up to 90%) but also significant relapses (up to 78%) [92, 93, 95–99]. In 2011, a multicenter retrospective study investigated the long-term outcome of RTX in 33 patients with CVID-associated refractory AC. RTX was usually given at standard dose, namely 375 mg/m^2^ once a week for 4 weeks. The authors found an overall initial response rate of 84% (with platelets > 30 × 10^3^/µl or Hb > 10 g/dl), including 74% complete responses (defined as platelets > 100 × 10^3^/µl or Hb > 12 g/dl). Durable response rate (complete response lasting more than 12 months) was observed in 59% patients. Major infections were the most frequent and severe adverse drug reaction (24.2%, 8/33) over a mean follow-up period of 39 months [19]. However, the incidence of major infections was not different from that observed in CVID patients with AC undergoing the first line of treatment, including glucocorticoids with or without high-dose IVIG [91].

In conclusion, RTX may be considered an effective therapeutic option for CVID-related AC, in particular in the subset of patients who cannot receive long-term high-dose glucocorticoids or present surgical contraindications (i.e., portal system hemodynamic changes) to splenectomy. Moreover, the effectiveness of B-cell depletion suggests that other molecules targeting B-cells (i.e., bortezomib, epratuzumab, ibrutinib) may also be effective in CVID-related AC [100–102].

### Granulomatous lymphocytic interstitial lung disease

A subset (5 to 22%) of CVID patients develop granulomatous lymphocytic interstitial lung disease (GLILD) [103–105]. The ethiopathogenetic features of this disorder are not completely understood and its characterization lies on both radiological abnormalities and histopathological findings [106]. Lymphocytic interstitial pneumonia (LIP), follicular bronchitis/bronchiolitis and granulomatous inflammation are the predominant histopathologic patterns, whereas the HR-CT finding of scattered nodules with areas of consolidation and ground glass, prominent on the lower zones, form the main radiographical pictures [107, 108].

Typical symptoms of GLILD are cough and dyspnea, while fever and pleuritic chest pain are rare. GLILD is frequently associated with splenomegaly, diffuse adenopathy, autoimmune cytopenias, and gastrointestinal and hepatic disease, thus suggesting a multi-systemic nature of the underlying pathogenesis. Various pathogenetic hypothesis have been proposed. Pulmonary granulomatous inflammation may develop in response to chronic antigenic stimulation, with pulmonary microenvironment preferentially favoring T-cell proliferation with a subsequent B-cell recruitment. On the other hand, the intrinsic immune dysregulation typical of CVID may favor the development of interstitial lung disease, as observed in CVID-like monogenic diseases such as cytotoxic T lymphocyte associated protein-4 (CTLA-4) deficiency and signal transducer and activator of transcription 3 (STAT3) gain-of-function mutations. Finally, a relatively high frequency of EBV, HIV and HHV-8 has been found in lung samples from patients with LIP, suggesting that viruses may play a role in triggering the lymphocyte infiltrate associated with GLILD [103–108]. To date, there is no established standard of care for the treatment of patients with CVID and GLILD, nor when it is necessary to start the treatment. These are important issues, since GLILD has been associated to a poor prognostic outcome. An early intervention targeting the polyclonal lymphocytic infiltration may reduce the rates of disability and mortality. Empirical treatments, including glucocorticoids, cyclosporine and infliximab, have been used with variable results. In the last 6 years, various case reports and small cohort studies have described the outcome of RTX, alone or in combination with classical immunosuppressants, in CVID patients with GLILD [62, 109–113].

The largest series included seven patients, who were treated with RTX at the weekly dose of 375 mg/m^2^ for 4 weeks (repeated at 4–6 month intervals, for 3 or 4 total courses) and oral azathioprine (1.0–2.0 mg/kg/day, 18 months duration) [62]. Post-treatment improvement was noted in both lung function (FEV1 and FVC) and radiographic findings (pulmonary consolidations, ground-glass, nodular opacities and bronchial wall thickening). No significant chemotherapy-related complications occurred. Similar results in terms of both effectiveness and safety were achieved in 4 case reports described between 2015 and 2017, by using the same therapeutic protocol including RTX and azathioprine [109–112]. In 2017 Jolles et al. described the use of 2-[(18)F]-fluoro-2-deoxy-d-glucose positron emission tomography and computed tomography (FDG PET-CT) scanning for the monitoring of response in a CVID patient with GLILD, treated with 2 infusions of RTX at dose of 1 g (with an interval of 1 month) in combination with long-term standard-dose (1–2 g/day) mycophenolate [113]. An almost complete resolution of the previously identified high metabolic activity and a normalization in lymph node size and lung architecture were observed. Recently, a RTX monotherapy was evaluated in 3 CVID naive patients: RTX was followed by an improvement in the severity and extent of both interstitial lung lesions and mediastinal adenopathy in all patients [20].

Collectively, these data confirm the effectiveness and safety of RTX in CVID-associated GLILD. On the other hand, a recent Consensus Statement on the management of GLILD in CVID, carried out by the British Lung Foundation/United Kingdom Primary Immunodeficiency Network, reports a 91% consensus on the use of glucocorticoids alone as first-line treatment [63]. Definitively, there is still a lack of longitudinal data on the long-term outcome and the impact on overall survival of the various therapeutic agents.

### Non-malignant lymphoproliferation and other conditions

A number of small series and case reports provide information about the effectiveness of RTX in various immune-mediated manifestations that are thought to be the result of the complex immune dysregulation of CVID. Salzer et al. described the successful outcome of RTX (375 mg/m^2^ administered weekly 4 times) to treat the systemic inflammatory response and lymphoproliferative disorder associated with EBV persistent replication in a pediatric patient with monogenic-CVID due to an autosomal recessive CD27 deficiency [114]. A comparable outcome was obtained, with the same RTX dose in combination with glucocorticoids, for an EBV-related lymphoproliferation in a patient with a monogenic form of CVID due to a frameshift mutation in the NFKB1 gene leading to NF-κB1 haploinsufficiency [115]. Similarly, RTX efficacy was reported in an adult patients with multiple monoclonal lymphoproliferative lesions [116].

Also granulomatous manifestations other than GLILD have been treated with RTX. A retrospective analysis of 59 CVID patients with granulomatous manifestations from the French DEFI cohort, found that standard dose RTX in combination with glucocorticoids, used in three patients, led to one complete and two partial responses of granulomatous lymph nodes and liver infiltrates [117].

Finally, the successful use of RTX have been reported in single cases of ANCA-associated vasculitis, Takayasu arteritis, renal granuloma, intra-cranial granulomata, bronchus-associated lymphoid tissue hyperplasia and cerebellar inflammatory lesions in patients with CVID [118–123].

## Conclusion

There is compelling evidence that autoimmune and lymphoproliferative disorders strongly affect the long-term outcome of CVID patients. The management of these complications often require multiple lines of treatment including various immunosuppressive drugs. Although glucocorticoids remain the first line of treatment for all the complications of CVID associated with the immune-dysregulation, over the last 5–10 years, RTX has proven to be an effective and relatively safe second-line therapy for both autoimmune and non-malignant lymphoproliferative manifestations. To date, there are no data on the long-term efficacy and side effects of RTX and other second-line therapeutic options. Further trials evaluating the head-to-head comparison between RTX and other second-line therapeutic options are needed to optimize the management strategies of non-infectious complications of CVID.

## Abbreviations

AC: autoimmune cytopenias
ADCC: antibody-dependent cell-mediated cytotoxicity
ADCP: antibody-dependent cell-mediated phagocytosis
AIHA: autoimmune hemolytic anemia
AN: autoimmune neutropenia
CVID: common variable immunodeficiency
EMA: European Medicines Agency
FDA: Food and Drug Administration
GLILD: granulomatous lymphocytic interstitial lung disease
IgRT: immunoglobulin replacement therapy
ITP: immune thrombocytopenia
IVIG: intravenous immunoglobulin
RTX: rituximab
